# Supplementary material for: A family-based intervention for prevention and self-management of disabilities due to leprosy, podoconiosis and lymphatic filariasis in Ethiopia: A proof of concept study
Source: PLoS Negl Trop Dis. 2021 Feb 18;15(2):e0009167. doi: 10.1371/journal.pntd.0009167 (PMC7924793; doi:10.1371/journal.pntd.0009167)
Supplement: S1 Text — (DOCX) [file pntd.0009167.s002.docx]

Supporting file information – Family-based intervention

The family-based intervention was delivered in several training sessions/group meetings and consisted of the following components:

1. Awareness raising
2. Disability management
3. Socio-economic empowerment.

**Format**
Several group meetings were held in a health centre close to the homes of the participants. These meetings or ‘sessions’ were delivered in group format (several families participated with one person affected and at least one family member present per family) to introduce the family-based intervention for self-management and prevention of disabilities.

**Disability management**
In the first ‘session’ basic training was given to persons affected and their family members in using and giving social support, increasing prevention and self-management of disabilities skills, information on course and treatment of disease, identifying barriers and facilitators to self-care and creating strategies to overcome these barriers. Participants also received basic tools to practice self-care (Vaseline, soap, a bucket and bandaging). In the following training sessions, the facilitators supported and guided all participating families (repeating the basic training given in the first session) and were available to clarify questions. Family members were encouraged to help their affected family member with self-care at home.

**Awareness raising**

A total of three flyers were prepared: one for each condition. In addition, three posters for leprosy, podoconiosis and lymphatic filariasis were prepared, encouraging people to seek treatment when they noticed early signs and symptoms of the diseases. These purpose of these posters and flyers were to teach the community and to spread awareness about the three conditions, we assumed that those able to read the materials would share the information with other community members. These materials were developed in collaboration with a delegate of the Ministry of Health. Because it took a long time to develop and pre-test the flyers, they were only disseminated to the participants after the intervention was completed. Basic information about the three conditions (e.g. symptoms, cause, treatment) was also given during the group sessions.

**Socio-economic empowerment**
Socio-economic empowerment consisted the formation of establishing Disabled People’s Organisations (DPOs). Two DPOs, the leprosy specific group in Addis Kidam and in Injibara, existed already (initiated by the Ethiopian National Association of Persons Affected by Leprosy/ENAPAL). A DPO for all three conditions was established in Zigem. Participants in the intervention were encouraged to join these DPOs. Each DPO collected a small contribution fee from its participants, 5 to 20 birr each month (less than one dollar or euro). These fees were used to provide loans for the participants (micro-finance). DPOs also lobbied for ‘benefits’, e.g. the use of land, from the government. These groups met monthly. While the facilitators of the project helped to establish these groups and were present during the meeting, they did not give any guidance on the management of the groups. This was done by persons affected themselves. Income generation is essential for sustainable self-management and prevention of disabilities: without income, self-care items such as Vaseline and shoes cannot be bought. Income generation benefits the whole family.
