# Supplementary material for: A family-based intervention for prevention and self-management of disabilities due to leprosy, podoconiosis and lymphatic filariasis in Ethiopia: A proof of concept study
Source: PLoS Negl Trop Dis. 2021 Feb 18;15(2):e0009167. doi: 10.1371/journal.pntd.0009167 (PMC7924793; doi:10.1371/journal.pntd.0009167)
Supplement: S2 Text — (DOCX) [file pntd.0009167.s003.docx]

**S3 Supporting information file – domain scores for the FQoL, SSS and SALSA**

**Family quality of life**

When looking at the five domains of the Beach Centre FQoL scale (family interaction, parenting, emotional wellbeing, physical wellbeing and disability-related support), there was a significant improvement in the domains emotional, physical and disability-related support for persons affected at baseline compared to follow-up (p<0.001, unequal variances t-test). The mean scores on these domains have improved over 17%. All five domains have significantly improved for family members at follow-up (p<0.001, unequal variances t-test). The domains with the biggest mean improvement (>40%) for family members are emotional, physical and disability-related support.

**Table 1. Domain scores for persons affected and family members on the FQoL scale.**

| *FQoL* | **Persons affected** | | | | **Family members** | | | |
| --- | --- | --- | --- | --- | --- | --- | --- | --- |
|  | Baseline (n=95), mean (95%CI) | Follow up (n=141), mean (95%CI) | Difference (%) | p-value^a^ | Baseline (n=117), mean (95%CI) | Follow up (n=78), mean (95%CI) | Difference (%) | p-value^a^ |
| Family interaction (range 6-30) | 21.8 (21.0-22.6) | 21.7 (20.9-22.5) | -0.1 (-0.5) | 0.943 | 19.1 (18.3-19.9) | 23.2 (22.5-24.0) | 4.1 (21.5) | 0.000 |
| Parenting (range 6-30) | 21.2 (20.3-22.1) | 22.2 (21.3-23.0) | 1.0 (4.7) | 0.114 | 19.2 (18.3-20.1) | 24.1 (23.4-24.9) | 4.9 (25.5) | 0.000 |
| Emotional wellbeing (range 4-20) | 8.9 (8.5-9.3) | 10.6 (10.1-11.1) | 1.7 (19.1) | 0.000 | 7.3 (6.8-7.7) | 11.0 (10.5-11.5) | 3.7 (50.7) | 0.000 |
| Physical wellbeing (range 5-25) | 12.8 (11.9-13.6) | 15.2 (14.6-15.8) | 2.4 (18.8) | 0.000 | 10.8 (10.2-11.4) | 16.0 (15.2-16.8) | 5.2 (48.1) | 0.000 |
| Disability-related support (range 4-20) | 12.3 (11.6-13.0) | 14.4 (13.8-15.0) | 2.1 (17.1) | 0.000 | 11.1 (10.5-11.7) | 15.6 (15.1-16.2) | 4.5 (40.5) | 0.000 |

^a^ Difference between baseline and follow-up scores, calculated using Welch's unequal variances t-test.

**Stigma**Mean (SARI) stigma scores for persons affected significantly decreased from 24.0 at baseline to 16.7 at follow-up. Mean scores on three out of the four domains of the SARI stigma scale significantly decreased after the intervention, these include experienced, internalised and anticipated stigma (p<0.05, unequal variances t-test). The mean difference on the domain disclosure decreased, but this difference was not significant (p<0.05, unequal variances t-test). An overview can be found in Table 2. Table 3 shows the domain scores per participant group.

**Table 2. Domain scores for persons affected (pooled) on the SARI stigma scale.**

| *SARI* | **Baseline (n=94), mean (95%CI)** | **Follow up (n=149), mean (95%CI)** | **Difference (%)** | **p-value^a^** |
| --- | --- | --- | --- | --- |
| Experienced stigma (range 7-21) | 8.3 (7.2-9.3) | 5.1 (4.4-5.8) | -3.2 (-38.6) | 0.000 |
| Disclosure concerns (range 4-12) | 4.4 (3.8-4.9) | 3.6 (3.1-4.1) | -0.8 (-18.2) | 0.054 |
| Internalised stigma (range 6-18) | 6.7 (6.0-7.4) | 4.5 (3.9-5.2) | -2.2 (-32.8) | 0.000 |
| Anticipated stigma (range 4-12) | 4.7 (4.2-5.3) | 3.5 (3.0-4.1) | -1.2 (-25.5) | 0.002 |

^a^ Difference between baseline and follow-up scores, calculated using Welch's unequal variances t-test.

**Table 3. Domain scores for persons affected by leprosy and persons affected by LF or podoconiosis on the SARI stigma scale.**

| *SARI* | **Persons affected by leprosy** | | | | **Persons affected by LF or podoconiosis** | | | |
| --- | --- | --- | --- | --- | --- | --- | --- | --- |
|  | Baseline (n=62), mean (95%CI) | Follow up (n=78), mean (95%CI) | Difference (%) | p-value^a^ | Baseline (n=31), mean (95%CI) | Follow up (n=71), mean (95%CI) | Difference (%) | p-value^a^ |
| Experienced stigma (range 7-21) | 7.5 (6.2-8.8) | 5.0 (3.9-6.1) | -2.5 (-33.3) | 0.004 | 9.5 (7.7-11.3) | 5.3 (4.4-6.1) | -4.2 (-44.2) | 0.000 |
| Disclosure concerns (range 4-12) | 4.0 (3.2-4.7) | 3.7 (2.8-4.5) | -0.3 (-7.5) | 0.592 | 5.1 (4.3-5.9) | 3.6 (2.9-4.3) | -1.5 (-29.4) | 0.004 |
| Internalised stigma (range 6-18) | 6.4 (5.4-7.4) | 4.5 (3.4-5.5) | -1.9 (-29.7) | 0.008 | 7.1 (6.2-8.0) | 4.6 (3.8-5.4) | -2.5 (-35.2) | 0.000 |
| Anticipated stigma (range 4-12) | 4.9 (4.1-5.6) | 3.4 (2.6-4.3) | -1.5 (-30.6) | 0.014 | 4.4 (3.6-5.1) | 3.6 (2.9-4.3) | -0.8 (-18.2) | 0.126 |

^a^ Difference between baseline and follow-up scores, calculated using Welch's unequal variances t-test.

**Activity limitations**

Table 4 shows the different categories of the SALSA (no, mild, moderate, severe or extreme activity limitations) and the number of participants in the in each category at baseline and follow-up. The change in the severe limitations group was the only significant difference between baseline and follow-up. The percentage of persons affected with severe limitations significantly decreased at follow-up (p<0.05, unequal variances t-test, pooled data of persons affected by leprosy, podoconiosis and LF).

**Table 4. The number of persons affected with no, mild, moderate, severe or extreme activity limitations on the SALSA scale pre- and post-intervention.**

| *SALSA* | **Baseline** | | | **Follow-up** | | | p-value^a^ |
| --- | --- | --- | --- | --- | --- | --- | --- |
|  | Persons affected by leprosy (n=43) | Persons affected by LF or podoconiosis (n=28) | **Total baseline** | Persons affected by leprosy (n=75) | Persons affected by LF or podoconiosis (n=51) | **Total follow-up** |  |
| **No (range 10-24)** | 3 | 5 | **8** | 6 | 4 | **10** | 0.459 |
| **Mild (range 25-39)** | 13 | 22 | **35** | 38 | 42 | **80** | 0.056 |
| **Moderate (range 40-49)** | 10 | 0 | **10** | 15 | 4 | **19** | 0.850 |
| **Severe (range 50-59)** | 14 | 0 | **14** | 8 | 1 | **9** | 0.019 |
| **Extreme (range 60-80)** | 3 | 1 | **4** | 8 | 0 | **8** | 0.839 |

^a^ Difference between baseline and follow-up scores (‘total baseline’ and ‘total follow-up’), calculated using Welch's unequal variances t-test.
