# Supplementary material for: A family-based intervention for prevention and self-management of disabilities due to leprosy, podoconiosis and lymphatic filariasis in Ethiopia: A proof of concept study
Source: PLoS Negl Trop Dis. 2021 Feb 18;15(2):e0009167. doi: 10.1371/journal.pntd.0009167 (PMC7924793; doi:10.1371/journal.pntd.0009167)
Supplement: Attachment — Submitted filename: Point-by-point reply_FBA.docx [file pntd.0009167.s004.docx]

**Point-by-point reply document

Manuscript number**: PNTD-D-20-01529 **Manuscript title**: “A family-based intervention for prevention and self-management of disabilities due to leprosy, podoconiosis and lymphatic filariasis in Ethiopia:  A proof of concept study”
**Deadline for submission of revision**: mid-December

| **Reviewer comment** | **Reaction authors** | **Changes in manuscript (highlighted in yellow)** |
| --- | --- | --- |
| There is a difference in the sample size determined and the total sample included in the study. Is the sample size calculated for family members or affected individual? Line 149-153: the calculated sample size is 30 families affected by leprosy  and 30 families of persons affected by LF or Podoconiosis. But the total sample included is larger than calculated. Why? *(and)* Line 258-262: When the calculated sample size is 30 family members each among people affected by leprosy and LF/Podoconiosis, why would the investigator included 275 participants. Any justification? | We thank the reviewer for this comment. We realise we have not explained this – we have now added our rationale for including more participant than initially planned/calculated. | The following was added to line 155-157: “To account for loss to follow up, we initially aimed to include at least 60 families in the intervention: 30 families of persons affected by leprosy and 30 families of persons affected by lymphatic filariasis or podoconiosis. However, because we anticipated that participants would benefit from the intervention, we decided to include as many participants as possible, so more families would benefit.” |
| Line 172-173: The sampling strategy to include people affected by leprosy appears to be flawed. Why didn’t they select participants (for people affected by leprosy) from lists available in the health post just like the strategy used to include LF/Podoconiosis (line 169-172). | We thank the reviewer for this comment. The persons affected by leprosy were indeed selected differently (contacted through organisations of persons affected by leprosy in the study areas). We have also mentioned non-random sampling as a limitation. We will ensure participants are selected in the same way when we conduct a (follow-up) randomised controlled trial. We have now added our reason for selecting the participants affected by leprosy differently. | The following has been added to line 177-180:  “Persons affected by leprosy were contacted through organisations of persons affected by leprosy in the study areas. This strategy was chosen because of the close connection of the Ethiopian National Association of Person Affected by Leprosy (ENAPAL), one of the organisations involved in the implementation of the intervention, with organisations of persons affected by leprosy in the study areas.” |
| Some concerns: The awareness raising intervention was not implemented as planned (line 351-352, 487-488) …..the printed materials were only distributed after the follow-up assessments had been conducted. This raises the question on attribution of awareness raising on outcome. Line 337-338 claim on awareness raising appears to be due to education give as part of self-management than awareness raising. | We thank the reviewer for this comment. Initially we wanted to both disseminate flyers and raise awareness/give education in the group sessions. Unfortunately only the latter was rolled out (Line 499-502: “In addition to providing education about self-care and clinical manifestations during the group sessions, in the present study it was initially also planned to distribute printed material in the communities, to raise awareness and reduce stigma. Due to time constraints, the printed materials were only distributed after the follow-up assessments had been conducted.”)   While we have identified ‘awareness raising’ as a separate component, health education and self-care training are often integrated in self-care and morbidity management training in other studies (also mentioned in our discussion on line 483-485). We have raised awareness as part of our intervention, but not as much as we hoped.  This component will be stronger in the RCT we are planning. We will also include a questionnaire to assess the impact of the awareness raising component then, that was not done in this study (e.g. a Knowledge Attitudes and Practices measure), this is also a recommendation in our discussion: “We recommend including pre- and post-intervention assessment of knowledge and community stigma in future studies, to get a better understanding of the changes in knowledge and community stigma.”  We note the comment of the reviewer with thanks and hope we have explained this sufficiently (also in the manuscript). | We have removed (line 511) “In the present study, stigma had significantly decreased after the intervention” to clarify that we do not link the reduction in stigma to the awareness raising component of the intervention. |
| Line 253-254: this is the intervention study, written consent would have been ideal. | We thank the reviewer for his/her comments, we fully agree. Given the low literacy rate of our study participants, we opted for verbal consent for this pilot intervention study. (Line 259-261 “Since the literacy rate was low in our study area, all participants were verbally informed about the nature and objective of the study, of confidentiality of the data and the voluntary nature of the study prior to data collection”) | - |
| Line 275 - typo - should be 'out of the 312 participants...' Line 278 - typo - '147' should be '179' (cf tabel 2) Line 320 - The text saus that the number who wore shoes decreaed - but in table 3 the number of people who wore shees increased (from 126 to 142) Line 518/519 - some impairments are permanent - amputations, loss of digits, fixed contractures etc. | We thank the reviewer for these helpful comments. This has been revised, we apologise for these typos/mistakes. | Line 275: “Out of the 275 participants who were invited to participate in the intervention,”  Line 278: “Follow-up data was collected of 219 participants for the FQoL (141 persons affected and 78 family members), 149 persons affected the SSS and 126 persons affected the SALSA” (149 refers to 78+71)  Line 320: “The number of persons affected by lymphatic filariasis and podoconiosis who had leg impairments and who had at least one acute attack per month ~~all~~ significantly decreased and who wore shoes significantly increased between baseline and participants’ last follow-up”  Line 518-519: “We believe the lack of improvement in eye impairments relates to their more chronic, permanent nature.”  Line 519-520: “We believe some of the hand impairments reported were either permanent (e.g. loss of digits) or may only have been reversed after a longer period of time,…” |
| Line 275-282: the description of table 2 is not clear.  Line 292-294: shows number of people in the follow-up has come down. Table 2 shows increase in number during follow-up?  Table 2: What is the justification for difference in number of participants interviewed for questionnaire between baseline and follow-up. *(and)* Line 275-282: the description is confusing. Should be simplified. | This has been revised. We apologise for any confusion, we hope we have clarified this by adding that the persons who were administered the questionnaires and interviewed, were a subset of those included in the intervention. A total of 275 persons were included in the first intervention session, this decreased over time. Follow-up interviews were done after the intervention and included a sample of the total people who were enrolled (275 in total, but not all attended all sessions, this decreased with each session). Slightly more participants were included in the follow-up data collection. We hope we have sufficiently clarified this. (We hope “Sometimes participants were for example present at the first, third and fourth session, but not at the second.” Line 295-296 helps clarify this) | Line 283-285 “Table 2 provides an overview” has been omitted. Instead, we have added the following to the beginning of the paragraph: “Table 2 provides an overview of the number of participants out of the total of 275 participants who were invited to participate in the study, who were administered the questionnaires and who were interviewed pre- and post-intervention. |
| Line 183-184: Simplified EHF score was measured to study the physical outcome, but not reported. Is Table 3 relating to EHF score?   Please provide reference for simplified EHF score Line 183-185: the data on simplified EHF score is not reported *(and)* Data collection - the simplified EHF - is possibly too simple and not clear. EHF gives us a range of 0-12. Is this simplified on a range of 0-3 or 0-6. That is, are you counting the hands together or seperately. If you are counting them together, then  avoid the term EHF score - as that is not what you have done. I see the small note under table 1, but the narrative could benefit from a small rewrite. | We thank the reviewer for this comment. We realise that by using the term ‘simplified EHF score’ we have caused confusion. We intended to use the EHF score, but due to miscommunication impairments were only recorded as present yes (1) or no (0), for eyes, hands and feet. We have adjusted this. | Line 183-185: “Physical impairment outcomes were assessed ~~using a simplified Eyes, Hands, Feet (EHF) score for leprosy (we scored impairments in as either no/0 or yes/1),”~~ by noting down if impairments were present for eyes, hands and feet for leprosy (we scored impairments as either not present/0 or present/1)…” |
| Line 320: people who wore shoes increased among people with LF and Podoconiosis Change in the shoe wearing among people affected by leprosy is not reported. | Unfortunately, we did not record whether persons affected by leprosy wore shoes or not.  (Line 183-190: “Physical impairment outcomes were assessed by noting down if impairments were present for eyes, hands and feet for leprosy (we scored impairments in as either not present/0 or present/1), and frequency of acute attacks, shoe wearing behaviour and foot and leg circumference for lymphatic filariasis and podoconiosis. Eye problems included …”) | - |
| Line 323-325: is the comparison between men and women for baseline measurement or at final follow-up, not clear. | This was calculated for the final measurement, using an independent samples t-test (Line 323-325 “Male participants had significantly larger leg circumferences on all four measurements (p<0.001 for foot and p<0.05 for leg circumferences, independent samples t-test).”) We now realise this information is not really relevant, we have therefore removed it from the manuscript. | Line 323-325 has been removed “Male participants had significantly larger leg circumferences on all four measurements (p<0.001 for foot and p<0.05 for leg circumferences, independent samples t-test).” |
| Table 4: Standard deviation or Standard Error to be provided for mean. If relevant, Median and IQR value can be provided. | This has been revised, the standard error (SE) has been added. | The standard error (SE) has been added to Table 4. |
| Line 436-437: is significant change in severe category for pooled data or for people affected by leprosy? | This has been clarified. | Line 436-437 “The percentage of persons affected with severe limitations significantly decreased at follow-up (p<0.05, unequal variances t-test, pooled data of persons affected by leprosy, podoconiosis and LF).” |
| Figure 1: A simple cross-tab comparing different categories of SALSA score for baseline and follow-up would be more useful than seeing the change in the percentage in individual category.  *(and)* Fig 1  - the Y axis will be clearer if the score range for each of the categories was included No = 10-24; Mild = 25-39; Mpderate = 40-49; Severe 50-59; Extreme 60-80.  (See <https://www.leprosy-information.org/media/627/download>) | We thank the reviewers for these comments. This has been revised. | Figure 1 has been removed. Instead, we have added a table with the sub-categories on the SALSA scale and p-values. We have added the range of each category also. We have added this table as supporting information file (S3) and removed it from the main text/manuscript, given the reviewers comments that the wording of our manuscript could be reduced. |
| The qualitative data analysis not provided | This is stated on line 245-247 “Qualitative data were analysed using Open Code 4.03 software. The qualitative data were transcribed and coded using open, inductive coding and content analysis. All data were anonymised before data analysis.” | - |
| Line 574-575: given the shorter duration of the intervention and the observation period it would be too ambitious to conclude as ‘impact’ of the intervention. Line 553-554 author says it is the short-term outputs. The observed change is the short-term outcome not impact. Another limitation is that the implementation of the intervention and outcome measurements were done by the same staff so possibility of measurement bias. | We have revised this. | Line 579-580: “This proof of concept study showed that the family-based intervention had a positive ~~impact~~ effect on impairments and self-management of disabilities, family quality of life and stigma”. This has been revised in line 53 also (conclusion of abstract). |
| The study is very interesting and presents a very useful kind of intervention to be implemented in other areas, and its design is adequate. I believe, however, that it can be presented, without losing information, in a more concise way. I believe that it is longer than it can be, and that its wording can be greatly reduced without undermining the ideas, the results and the discussion and reasoning that the authors want to convey to the reader. This is especially recommended in the chapter "Discussion". With regard to table n1, consider reducing the list of "sessions attended" by making groups (1; 2-4; 5-8). | We thank the reviewer for this comment. This has been revised. | We have cut down some of the text. Figure 1 has been removed from the manuscript and added as supporting information file (S3). In addition, in order to cut down some of the text, we have replaced ‘lymphatic filariasis’ with LF in the manuscript (except for the Tables). |
| Line 33: reorder the the words to 'activity limitations, stigma and family quality of care' to match the order the tools are listed | This has been revised | Line 33 “…data on ~~stigma,~~ activity limitations**,** stigma and family quality of life using…” |
| line 184 - typo remove 'in' | This has been revised | “in” has been removed (line 184). |
| Line 22-23: Not all patients with leprosy and LF will require life-long need to practice self-management… only those with impairment would need life-long care | We thank the reviewer for this comment. This has been revised. | “A key issue for persons with ~~affected by~~ leprosy-, lymphatic filariasis- and podoconiosis-related disabilities is the life-long need to practice self-management routines” (also in line 79-80) |
| Line 41: disability management.. elsewhere in the manuscript it is mentioned as self-management. Consistent use of term would be very helpful | We have used “self-management of disabilities” and “disability management” – we have now only used “self-management”. | Line 41, 117, 122, 479, 481, 482: We have revised “disability management” to “self-management of disabilities), |
| Line 75-55: Ref 16 is for the heart condition | Apologies, this has been revised. | Ref 16 has been omitted. |
| Line 455, 459: New aspects of our intervention are its integrated approach and ……. meaning of integrated is not clear? *(and)* The word integrated appears in the discussion. The author should be clear on what they meant by integrated. Are they meaning bringing affected person and family together as integrated or between the disease group? | This has been revised. | “Integrated” has been removed. |
| Line 165: Persons unwilling… is not an exclusion criteria | This has been revised. | “Unwilling” has been removed (“Persons ~~unwilling or~~ unable to give informed consent and persons younger than 15 years of age were excluded”) |
| Line 197, 214: are they same as study participants or different people? | We thank the reviewer for his/her comment. These were different participants. We have clarified this. | “The SSS was pilot tested among 15 participants (who did not participate in the pilot test of the FQoL) before use.” |
| Line 208: the word “assesses” | This has been revised. | “assesses” has been removed |
| Line 304: 95% CIs to be provided for odds ratio *(and)* Line 303-304: the reported relationship between age with post intervention physical outcome is not clear. The 95CIs should be given for the odds ratio. | This has been revised. | Line 304 “In addition, logistic regression showed that as age increased, the odds of having eye impairments also increased (p<0.05, odds ratio 1.047 with 95%CI 1.006-1.091).” |
| Line 392-398 and 401-407: Table not provided for the results explained here  (*and)* Line 392-398 & 401-407: data of sub scores to be provided for easy understanding | This has been revised. | We have now added these tables as supporting information file (S3). We have added this as supporting information file and not in the main text/manuscript given the reviewers comments that the wording of our manuscript could be reduced. |
| Line 502-506: author appears to be indicating that they have increased the income through socio-economic intervention, if so, the data should be added *(and)* Line 355-364: DPOs for people affected by leprosy already existed. No data on socio-economic intervention provided through this research is provided to justify its effect on the outcome. | We thank the reviewer for this comment. Although the DPOs did collect money that could be used as loans, we did not routinely collect information about how and if this was used (unfortunately). In our discussion, we believe we did not indicate or make any explicit statements about increasing income (because this information is unfortunately not available). However, in line 502-506 we have tried to emphasize the importance of socio-economic empowerment (“Sometimes, income is too low to acquire basic materials for self-care [46,48]. Costs for treatment, associated non-medical costs and reduced ability to work may cause a financial burden on the entire household of persons affected [5,18,49,50]. We therefore consider socio-economic empowerment an essential component of interventions for self-management and prevention of disabilities. Without income, self-care items such as Vaseline and shoes cannot be bought.” We hope we have done this accurately. We have added a line in the discussion about this. | We have added, line 518-519: “Unfortunately, we did not collect any information on how much money was collected by the DPOs as monthly fees, and if any loans were provided.” |
| Line 515-516: the explanation of odds ratio is not clear. | This has been revised. | We have removed this from the discussion (also in order to shorten the text of our manuscript). |
| I am struggling to really comprehend what is unique about FBA. what are the family members contributing to the gorup functioning and dynamics? Apart from the presence of family members, what is different abotu FBA to 'traditional' self-care groups? I think a few more sentences making this clear would strengthen the paper. | We thank the reviewer for his/her comments. We have tried to explain the added benefit on lines 79-89: “A key issue for persons with leprosy-, lymphatic filariasis- and podoconiosis-related disabilities is the life-long need to practice such self-management routines. This is difficult to sustain without regular encouragement and support of others. A strategy shown to be successful is the formation of self-care groups in which persons affected by leprosy, lymphatic filariasis and podoconiosis-related impairments support each other [18–20]. Self-care groups however, often have limited long-term sustainability and members may have problems in accessing the groups, for example because of financial or geographical barriers [19]. Family-based support may be a more sustainable and feasible strategy to practice self-management routines. Several studies have indicated that family support is a highly significant factor in adherence to self-care [21,22]. …”  We have also stressed this in our discussion (under ‘family-based format’), e.g. line 482-388: “…These challenges do not apply to family-based interventions, where participants do not need to travel to attend and no facilitator is needed. In addition, a family-based intervention is relatively inexpensive since this can be practiced at home and no travel is required. Furthermore, encouragement by significant others can increase motivation, which is essential for self-care behaviour [23,47]. Family-based support therefore seems like a sustainable option, especially in contexts where resources are scarce, which is often the case in areas where leprosy, lymphatic filariasis and podoconiosis are endemic [17,19,48–50]”   We agree with the reviewer that the main difference from traditional self-care groups is the presence of family members, and the encouragement of family members for self-care at home. Other reasons are listed above. We consider a family-based approach a (sustainable) alternative to traditional self-care groups / another possible option. We hope we have clarified this sufficiently. In the RCT we are planning, we hope to get a better understanding of the added benefit of the family members (by comparing the approach with ‘usual practice and care’). | - |
| I also have a question about sustainability of this 'proof of concept' - when there is a heavy investment of vaseline, bucket, soap and bandages. Does thes supply of these things counfound the results? | We thank the reviewer for this interesting point. Although we believe the investment of Vaseline and soap may have has some impact on for example quality of life, we do not believe it has had an impact on our primary outcome (physical impairments) as such. We do recognize that this is a limitation, we hope to address this in the RCT we are planning (in which both the intervention and the control group will be given Vaseline, soap and a bucket). | - |
| The logistic regression and linear regression are mentioned in the analysis plan but not reported. | We thank the reviewer for this comment. We have now clarified this in the methods section. We have omitted the linear regression of age and gender with leg circumference (we believe this is outcome does give us meaningful information).   In addition, we have reported on line 309-310: “In addition, logistic regression showed that as age increased, the odds of having eye impairments also increased (p<0.05, odds ratio 1.047).” Since there was no relationship between age and gender and number of acute attacks, shoe wearing, and hand and foot impairments at final assessment, we did not report it. However, we have now added this information. We thank the reviewer for this comment. | Added line 312-314: “There was no relationship between age and gender and number of acute attacks, shoe wearing behaviour, and hand and foot impairments and between gender and eye impairments at final assessment (p>0.05, logistic regression).  We have removed the linear regression from methods > data analysis. Line 241: “~~Linear regression and~~ Binary logistic regression ~~were~~ was performed to see if there was a relationship between gender and age and physical impairment outcomes at final measurement (number of acute attacks, shoe wearing behaviour and hand, eye and foot impairments)” |
